# Catheter Ablation in Atrial Fibrillation: Recent Advances

**DOI:** 10.3390/jcm13247700

**Published:** 2024-12-17

**Authors:** Sahib Singh, Lohit Garg, Mohammed Y. Kanjwal, Kevin Bliden, Udaya S. Tantry, Paul A. Gurbel, M. Chadi Alraies, Abdulla A. Damluji

**Affiliations:** 1 Department of Medicine, Sinai Hospital of Baltimore, Baltimore, MD 21215, USA; 2 Division of Cardiology, University of Colorado, Aurora, CO 80045, USA; garg.medicine@gmail.com; 3 Division of Cardiology, Sinai Hospital of Baltimore, Baltimore, MD 21215, USA; mkanjwal@lifebridgehealth.org (M.Y.K.); pgurbel@lifebridgehealth.org (P.A.G.); 4 Sinai Center for Thrombosis Research, Sinai Hospital of Baltimore, Baltimore, MD 21215, USA; kbliden@lifebridgehealth.org (K.B.); utantry@lifebridgehealth.org (U.S.T.); 5 Division of Cardiology, Wayne State University/Detroit Medical Center, Detroit, MI 48201, USA; alraies@hotmail.com; 6 Division of Cardiology, Inova Center of Outcomes Research, Falls Church, VA 22042, USA; abdulla.damluji@jhu.edu; 7 Division of Cardiology, Johns Hopkins University School of Medicine, Baltimore, MD 21287, USA

**Keywords:** atrial fibrillation, catheter ablation, radiofrequency, cryoballoon, pulsed field, mapping

## Abstract

Atrial fibrillation (AF) is the leading cause of arrhythmia-related morbidity and mortality. Recurrent symptoms, hospitalizations, and cost burden to patients have necessitated treatments beyond antiarrhythmic drugs (AADs) for patients with AF. Catheter ablation has proven to be effective over medical therapy alone; however the recurrence rates for atrial tachyarrhythmias post-ablation remain significant, particularly in patients with persistent and long-standing persistent AF. Hence, new techniques for catheter ablation have arisen, such as non-thermal energy sources, novel catheters, electroanatomical mapping, and ablation of additional targets. In this review, we discuss the recent advances in the field of catheter ablation, including newer modalities for the prevention of adverse events and future perspectives.

## 1. Introduction

Atrial fibrillation (AF) is one of the most common cardiac arrhythmias, with a prevalence of around 5.2 million (2010) in the United States (U.S.), which is projected to increase by up to 12.1 million by 2030 [1]. The estimated lifetime risk of developing AF is about 36% in white males, 30% in white females, 21% in black males, and 22% in black females [1]. AF is associated with a wide range of complications such as systemic embolic events, stroke (4- to 5-fold increased risk), heart failure (HF), recurrent hospitalizations and mortality. Adequate treatment and control of AF is important to prevent the complications and associated cost burden on patients and healthcare.

Rate and rhythm control medications are usually the first-line approach in patients with AF [2]. Catheter ablation of AF has been recommended for patients who remain symptomatic despite medical treatment, with the possible consideration of first-line management in paroxysmal AF [3,4,5,6,7,8,9,10]. The rate of freedom from atrial arrhythmia after ablation is around 50.6% (single ablation) and 69.7% (multiple ablations) at 5 years [11]. Repeat ablation for patients with recurrent AF has fewer clear guidelines, with limited available evidence compared to those undergoing de novo catheter ablation [12,13,14,15,16]. While pulmonary vein isolation (PVI) remains the primary type of catheter ablation in AF, several advances have been made in catheter designs, energy sources, mapping, and alternate ablation targets [17,18,19,20,21,22,23]. Additionally, innovative methods to prevent adverse events, such as esophageal injury, have been studied. Among the various AF types, patients with persistent and long-standing persistent AF have lower success rates with ablation, with a possible impact on diagnosis-to-ablation time (DAT) as well [24,25,26,27]. In this review, we discuss the current guidelines, recent studies on catheter ablation (from major online databases such as Cochrane, Pubmed, MEDLINE, and Embase), and future perspectives for the management of AF.

## 2. Clinical Guidelines

The 2023 American College of Cardiology (ACC)/American Heart Association (AHA) guidelines recommended: (1) Catheter ablation for symptomatic AF in patients with incomplete response or contraindications to antiarrhythmic drugs (AAD) (class 1); (2) Catheter ablation as first-line therapy in select patients (young age and few comorbidities) with symptomatic paroxysmal AF (class 1); (3) PVI as the primary target, unless other triggers are identified (class 1); (4) Value of ablating additional targets such as posterior wall, low voltage areas (LVA) and rotors is uncertain (class 2b); (5) Repeat ablation or AADs for recurrent symptomatic AF after catheter ablation (class 1); (6) Short-term AAD treatment after ablation to reduce the chances of recurrence (class 2a); (7) Concomitant surgical ablation can be considered in patients undergoing cardiac surgery (class 2a); (8) Hybrid epicardial/endocardial ablation for symptomatic persistent AF refractory to medical therapy (class 2b); (9) Catheter ablation in patients with AF and HF with a reduced ejection fraction (HFrEF) for improving symptom control, ventricular function, quality of life (QOL), and cardiovascular (CV) outcomes (class 1); (10) Catheter ablation to improve symptoms and QOL in patients with HF and preserved ejection fraction (HFpEF) (class 2a) [28].

The European Heart Rhythm Association (EHRA)/Heart Rhythm Society (HRS) released the expert consensus statement on AF ablation recently (2024), with the following notable additions: (1) It may be reasonable to consider catheter ablation as first-line treatment in symptomatic persistent AF (area of uncertainty); (2) Asymptomatic patients with recurrent paroxysmal or persistent AF may be candidates for catheter ablation after thorough discussion of the risks and benefits (area of uncertainty); (3) Catheter ablation may be appropriate for older patients (>75 years) and those with hypertrophic cardiomyopathy (HCM), taking into account the comorbidities and procedural efficacy; (4) Use of an esophageal temperature probe during thermal AF ablation to help guide energy delivery (area of uncertainty); (5) For persistent AF, ethanol infusion in the Vein of Marshal (EIVOM), mapping and ablation of non-PV triggers, and left atrial posterior wall (LAPW) isolation may be reasonable (area of uncertainty); and (6) Concomitant surgical ablation for patients with paroxysmal or persistent AF undergoing cardiac surgery irrespective of previous AAD failure or intolerance (Advice TO DO) [29]. The EHRA also published guidance on CV imaging in AF patients undergoing ablation, with consideration of cardiac-computed tomography (CT) or magnetic resonance (MR) to determine left atrial appendage (LAA) morphology, LA and PV anatomy, and to rule out LAA thrombus [30].

## 3. Radiofrequency Ablation

PVI using radiofrequency ablation (RFA) was the classical therapeutic option used earlier, initially with moderate power bursts (25–35 W over 30–60 s) and then with contact force (CF)–sensing catheters (Table 1) [31]. Subsequently, a high-power, short-duration (HPSD) technique was developed for RFA with potentially faster ablation using resistive heating (40–50 W over a shorter time) [32]. The High Power-Short Duration Versus Standard Power-Long Duration Radiofrequency Ablation for Treatment of Atrial Fibrillation (SHORT-AF) trial randomized 60 patients with paroxysmal or persistent (<1 year) AF to HPSD (50 W) or standard power, standard duration (SPSD, 25–30 W) RFA for PVI (Table 2) [31]. The HPSD group was associated with a shorter time to achieve PVI (87 vs. 126 min, *p* = 0.003) and LA dwell time (157 vs. 180 min, *p* = 0.04), without any differences in first-pass isolation (79% vs. 76%, *p* = 0.65) or PV reconnection with adenosine (12% vs. 20%, *p* = 0.26). Although the HPSD group had less recurrent atrial arrhythmias at 12 months (10% vs. 35%, *p* = 0.027), it showed a higher trend of asymptomatic cerebral emboli (40% vs. 17%, *p* = 0.053). Going a step further, the higher power–short duration vs. lower power longer duration posterior wall ablation for atrial fibrillation and oesophageal injury outcomes (Hi-Lo HEAT) trial reported similar incidences of oesophageal thermal injury (*p* = 1.0) and maximal oesophageal temperature (38.6 °C vs. 38.7 °C, *p* = 0.43) in the two groups [33]. Combining data from six randomized studies (694 patients), the meta-analysis by Amin et al. showed that the HPSD group had a decreased total procedure time (*p* = 0.0007), PVI time (*p* < 0.00001), and RF time (*p* < 0.00001), with a similar incidence of esophageal lesions (*p* = 0.77) and first-pass isolation (*p* = 0.65) when compared with the SPSD group [34].

Based on the same concept as HPSD, very high-power short-duration (vHPSD) ablation (around 90 W for 4 s) has also been studied [35,36,37]. The Very High Power Ablation in Patients With Atrial Fibrillation Schedule for a First Pulmonary Vein Isolation (POWER PLUS) trial randomized 180 patients to 90-W/4-s ablation and 35/50-W ablation groups [38]. The vHPSD group was associated with shorter procedural time (70 vs. 75 min, *p* = 0.009), with no significant differences in first-pass isolation rate (83.9% vs. 90%, *p* = 0.0852), esophageal injury (1 patient per group) and recurrent arrhythmias at 6 months (17% vs. 15%, *p* = 0.681). Tokavanich et al. conducted a network meta-analysis of vHPSD, HPSD and conventional power ablation (29 studies, 9721 patients) [39]. Both vHPSD and HPSD groups had faster procedure times than conventional ablation; however the HPSD ablation group had the highest probability of maintaining sinus rhythm and first-pass isolation.

Among the other novel catheters being tested, Hussein et al. reported that ablation using the temperature-controlled RF catheter QDOT MICRO (QDM; Biosense Webster, Inc., Irvine, CA, USA) resulted in a clinically meaningful improvement in QOL along with a significant reduction in AAD use, cardioversion, and CV hospitalization when compared to baseline characteristics [40]. Solimene et al. used CF-localized impedance (LI)-guided PVI in 275 AF patients, with a success rate of 100%, minor complications in 4.4% and AF recurrence in 13.1% at 1 year [41].

## 4. Cryoablation

Cryoballoon ablation (CBA) or cryoablation has emerged as an effective alternative to RFA for catheter ablation in AF. The 3-year follow up of the Early Aggressive Invasive Intervention for Atrial Fibrillation (EARLY-AF) trial showed that initial rhythm-control therapy with CBA was superior to AAD therapy in terms of lower incidence of persistent AF (1.9% vs. 7.4%), recurrent atrial tachyarrhythmias (56.5% vs. 77.2%) and hospitalizations (5.2% vs. 16.8%) [42]. Zucchelli et al. reported outcomes from the Cryo Global Registry: as compared to CBA in AAD-refractory patients, first-line CBA was associated with greater mean reduction in symptoms and prescription of AADs at 12 months (9.7 vs. 29.9%), without any difference in serious procedure-related adverse events, efficacy (when controlling for baseline characteristics, *p* = 0.55), repeat ablations, hospitalizations, and QOL (*p* = 0.29) [43].

One of the most common CBA-related complications is right-sided phrenic nerve injury (PNI) which leads to the premature abortion of the freeze cycle, resulting in around 24.7% of initially isolated index PVs to show reconnection during repeat ablations [44]. Several new iterations and modifications of CBA are being evaluated to enhance its safety and efficacy [45,46]. In the randomized trial by Ahn et al., fluoroless CBA for AF under intracardiac echocardiography (ICE) guidance (Zero-X) was compared with the conventional group [47]. The two groups had comparable procedure time, LA indwelling time, complication rate and recurrence rate (*p* = 0.841). De Greef et al. demonstrated that the addition of electroanatomical EnSite mapping to CBA resulted in a lower incomplete PVI rate compared to conventional CBA (0 vs. 12%, *p* = 0.012) [48]. Along similar lines, Wubulikasimu et al. reported results of a new three-dimensional (3D) mapping system KODEX-EPD used to guide CBA, which reduced fluoroscopy time (532.30 s vs. 676.25 s, *p* < 0.05), fluoroscopy dose (110.00 mGy vs. 144.68 mGy, *p* < 0.05) and contrast medium dosage (71.90 mL vs. 76.05 mL, *p* < 0.05) when compared with the conventional two-dimensional perspective group [49].

Comparison of RFA and CBA has been assessed in multiple studies [50,51,52]. In the randomized study of patients with persistent AF by Mililis et al., RFA and CBA had similar risk of recurrences ≤ 3 months (35.5% vs. 37.9%, *p* = 0.755) and >3 months (26.3% vs. 27.3%, *p* = 0.999), with RFA having a longer procedure duration (136.6 vs. 75.15, *p* < 0.05) [53]. On the other hand, Andrade et al. demonstrated that patients with drug-refractory paroxysmal AF had lesser progression to persistent AF when treated with CF-guided RFA (0%) with 4 min CBA (7%) or 2 min CBA (4.3%, *p* = 0.03) [54]. In the meta-analysis by Kim et al., CBA had a shorter procedure time (mean reduction 43.77 min) but greater risk of PNI (risk ratio [RR] 4.13) than RFA, without any differences in freedom from recurrent atrial tachyarrhythmia, total complications, and fluoroscopy time [55].

## 5. Pulsed Field Ablation

As opposed to the conventional thermal energy ablations (RFA/CBA), pulsed field ablation (PFA) is a nonthermal approach which uses high-voltage electrical fields to create nanopores in the cardiac cellular membrane, causing cellular necrosis [56,57,58]. The Randomized Controlled Trial for Pulsed Field Ablation versus Standard of Care Ablation for Paroxysmal Atrial Fibrillation (ADVENT) showed noninferiority of PFA to conventional thermal ablation in terms of freedom from a composite of initial procedural failure, documented atrial tachyarrhythmia after a 3-month blanking period, antiarrhythmic drug use, cardioversion, or repeat ablation (73.3% vs. 71.3%), and device- and procedure-related serious adverse events (2.1% vs. 1.5%) [59,60,61]. Using a variable-loop circular catheter integrated with a 3D mapping system, De Potter et al. reported the optimal number of PFA applications to be ≥48 (total) or ≥12 (per vein) for achieving a 1-year success rate of around 80% [62]. Among the meta-analyses comparing PFA to thermal ablation, de Campos et al. included 18 studies (4998 patients) and found that PFA was associated with a lesser procedure time (mean difference [MD] −21.68), esophageal injury rate (odds ratio [OR] 0.17) and treatment failure rate (OR 0.83), along with a longer fluoroscopy time (MD 4.53), higher tamponade rate (OR 2.98) and better first-pass isolation rate (OR 6.82) [63,64].

**Table 2:** Major clinical trials.

Study	Energy Type	Sample Size	Procedure Time (Minutes)	Complications	Freedom from Atrial Tachyarrhythmia	Follow Up
SHORT-AF [31]	HPSD RFA vs. SPSD RFA	29 31	236 (193–304) 286 (219–350)	1 1	HR 0.26 (*p* = 0.027)	12 months
POWER PLUS [38]	35–50 W RFA vs. 90 W RFA	90 90	75 (65–88.3) 70 (60–80)	1 1	Recurrence (15% vs. 17%)	6 months
EARLY-AF [42]	CBA vs. drug therapy	154 149	-	17 35	Recurrence (56.5% vs. 77.2%)	36 months
ADVENT [59]	PFA vs. Thermal Ablation	305 302	105.8 ± 29.4 123.1 ± 42.1	7 6	Recurrence (17.2% vs. 16.4%)	1 year

HPSD—high power, short duration, SPSD—standard power, standard duration, HR—hazard ratio, RFA—radiofrequency ablation, CBA—cryoballoon ablation, PFA—pulsed field ablation.

Advances in PFA include the development of a lattice-tip catheter which has a larger footprint, enabling faster PVI with fewer required applications [65,66]. Reddy et al. recently reported the first in-human study of a lattice single-shot PFA catheter along with a dedicated electroanatomical mapping system [67]. PVI was achieved in 100% of PVs, with a procedure time of around 56.5 ± 21.6 min, fluoroscopy time of 5.7 ± 3.9 min, no primary safety events, and 1-year freedom from atrial arrhythmias in 81.8% of cases. Sanders et al. conducted a study of the balloon-in-basket, 3D-integrated PFA system, which was effective in 96.9% patients, with the procedure time of 124.6 ± 28.1 min and fluoroscopy time of 19.8 ± 8.9 min [68]. No PNI, PV stenosis, oesophageal lesions or primary serious adverse events were reported. Similar promising results have been shown with the use of a multi-electrode hexaspline PFA catheter, ICE-guided PFA and variable-loop PFA catheter integrated with an electroanatomic mapping system as well [69,70,71]. Lastly, deep sedation protocols are being tested which could offer alternatives to general anesthesia during PFA [72,73].

It should be noted that in PFA, there is no class effect. Unlike thermal energy ablation, each device uses its own “formula” for the number, duration, amplitude, and interval of pulses. Currently, the majority of available data comes from the pentaspline PFA ablation system. However, due to the above-mentioned biophysical differences, these findings cannot be generalized to other PFA systems.

## 6. Additional Targets

Although PVI is the standard approach in patients with AF, recurrences are common, necessitating the need for other ablation targets and intervention on the pro-fibrillatory substrate [74,75,76,77,78,79,80,81,82,83,84,85,86,87,88]. The Catheter Ablation for Persistent Atrial Fibrillation: A Multicenter Randomized Trial of Pulmonary Vein Isolation vs. PVI With Posterior Left Atrial Wall Isolation (CAPLA) study showed that the two strategies were comparable with respect to freedom from recurrent atrial arrhythmia without AAD after a single procedure (53.6% vs. 52.4%, *p* = 0.98), freedom from atrial arrhythmia with/without AAD after multiple procedures (60.1% vs. 58.2%, *p* = 0.57), and freedom from symptomatic AF with/without AAD after multiple procedures (72% vs. 68.2%, *p* = 0.36) [89]. The lack of benefit from additional LAPW isolation was also demonstrated in patients with posterior LVAs [90]. However, in the CAPLA substudy of patients with rapid LAPW activity, the PVI + LAPW isolation group had greater arrhythmia-free survival than PVI alone (56.4% vs. 38.6%, *p* = 0.030) [91]. In the meta-analysis of 10 studies by Shrestha et al., PVI + LAPW ablation was associated with lower recurrence of overall atrial tachyarrhythmias (OR 0.47) and AF (OR 0.39), with greater freedom from atrial arrhythmias (OR 2.22), without any difference in atrial flutter incidence (OR 1.36) or periprocedural adverse events (OR 1.10) [92].

Among the meta-analyses evaluating efficacy of adjunctive LVA ablation, Rivera et al. pooled data from 10 randomized trials (1780 patients) showing that adjunctive LVA ablation was associated with a reduction in atrial tachyarrhythmia recurrence (RR 0.76, *p* < 0.01), especially in those with documented LVAs on baseline substrate mapping (RR 0.57, *p* < 0.01), along with a reduction in redo ablation procedures (RR 0.54, *p* < 0.01) when compared with conventional ablation [93,94,95,96]. Rackley et al. conducted a meta-analysis of five randomized studies comparing PVI with adjunctive ganglionic plexus ablation to endocardial catheter-based PVI, and found the former group to have lower odds of arrhythmia recurrence (OR 0.58), particularly in those with paroxysmal AF (OR 0.396) [97]. The Augmented Wide Area Circumferential Catheter Ablation for Reduction of Atrial Fibrillation Recurrence (AWARE) study compared conventional adenosine-guided wide-area circumferential ablation (WACA) with a novel augmented (double) WACA technique [98]. Recurrent atrial arrhythmias at 1 year (26.7% vs. 24.6%, *p* = 0.64), repeated catheter ablation (10.3% vs. 7.4%), and serious adverse events (6.7% vs. 6.9%) were comparable in the two groups.

## 7. Mapping

Electroanatomical mapping allows for the localization of focal activation patterns during AF, which can potentially serve as targets for ablation. The Mapping-Guided Ablation for Persistent Atrial Fibrillation (MAP-AF) trial randomly assigned patients to PVI with mapping-guided ablation of focal activation sites (n = 98) vs. PVI alone (n = 102) [99]. No significant differences were found in the two groups with respect to freedom from AF/atrial tachycardia without AADs at 2 years (66.8% vs. 75.2%, *p* = 0.37) and adverse events (3.0% vs. 0%, *p* = 0.12). Vandenberk et al. conducted a prospective study utilizing high-density (HD) mapping (HD Grid) of the PVs to identify the incidence of conduction gaps in the RFA lesions after PVI and compared them with a standard 20-pole circumferential mapping catheter (CMC-20) [100]. Although the mapping time was similar in the HD Grid and CMC-20 groups (11.7 ± 3.4 min vs. 12.2 ± 2.6 min, *p* = 0.452), the former was associated with a significantly higher number of points (1662.7 ± 366.1 vs. 1171.6 ± 313.6, *p* < 0.001).

In a retrospective study by Dittrich et al., omnipolar mapping (OT), which displays true voltage and real-time wavefront direction and speed independent of catheter orientation, was compared against standard bipolar settings (SD) and HD wave (HDW) algorithm [101]. Among the 30 patients treated for LA arrhythmia, using OT showed higher point densities (21,471 vs. 6682 SD vs. 12,189 HDW, *p* < 0.001), higher mean voltage (0.75 mV vs. 0.61 mV [SD] vs. 0.64 mV [HDW], *p* < 0.001), and detected more PV gaps per patient (4 vs. 2 SD, *p* = 0.001). Similar trends were noticed in LV maps, where OT showed higher point densities (25,951 vs. 8582 SD vs. 17,071 HDW, *p* < 0.001), higher mean voltage (1.49 mV vs. 1.19 mV [SD] vs. 1.2 mV [HDW], *p* < 0.001) and smaller detected scar area (25.3% vs. 33.9% SD, *p* < 0.001). Dong et al. conducted a randomized trial evaluating electroanatomical mapping-guided superior vena cava isolation (SVCI) in addition to circumferential PVI vs. circumferential PVI alone in paroxysmal AF patients without provoked SVC triggers, and found the two groups to be similar regarding freedom from atrial tachyarrhythmias (87.9% vs. 79.6%, *p* = 0.28) [102]. In the randomized study by Lin et al., periodicity and similarity (PRISM) mapping added to PVI demonstrated higher freedom from AF at 12 months (70.6% vs. 47.1%), when compared with conventional ablation [103]. Similar trends of variable results were observed in other recent studies as well, highlighting the developing nature of different mapping algorithms in AF ablation [104,105].

## 8. Long-Standing Persistent AF

While catheter ablation is well established for paroxysmal AF, the outcomes have been suboptimal for patients with persistent and long-standing persistent AF. A retrospective study reported that a preprocedural average heart rate ≥ 85 beats/min in patients with long-standing persistent AF was independently associated with the maintenance of sinus rhythm after RFA (OR 3.30, *p* = 0.003) [106]. Other studies showed LA contractile strain at 3 months and amplified P-wave (APW) duration to be predictors of AF recurrence following ablation of long-standing persistent AF [107,108,109]. Various combinations and novel techniques are being evaluated to improve the success rates of long-standing persistent AF ablation [110,111,112,113,114,115,116,117,118,119].

The subanalysis of the Convergence of Epicardial and Endocardial Ablation for the Treatment of Symptomatic Persistent Atrial Fibrillation (CONVERGE) trial assessed hybrid vs. endocardial ablation in the long-standing persistent AF subgroup [120]. Freedom from atrial arrhythmias off AADs (new or increased dose of previously failed or intolerant) was higher in the hybrid ablation group at 12 months (65.8% vs. 37.0%, *p* = 0.022) and 18 months (60.5% vs. 25.9%, *p* = 0.006). Going a step ahead, Jiang et al. conducted a propensity-matched cohort study of long-standing persistent AF patients undergoing concurrent hybrid ablation (catheter ablation and left unilateral thoracoscopic epicardial ablation in one procedure) vs. staged hybrid ablation (epicardial ablation in the first hospitalization, with catheter ablation being reserved for those with AF recurrence) [121]. The groups had a similar rate of freedom from AF or atrial tachycardia off AADs at 2 years (79.9% ± 5.7% concurrent group vs. 86.0% ± 4.9% staged group, *p* = 0.390). Pooling the data from three randomized trials (358 patients), Rivera et al. conducted a meta-analysis and trial sequential analysis demonstrating that hybrid ablation was associated with a reduction in the recurrence of atrial tachyarrhythmias (RR 0.53, *p* < 0.01), without any significant difference in major adverse events (RR 1.22, *p* = 0.68), when compared with endocardial ablation for patients with persistent/long-standing persistent AF [122]. Modifications in hybrid ablation, such as robotic-enhanced epicardial ablation and adjunctive LAA exclusion, have shown promising results as well [123,124].

Bianchini et al. compared hybrid RFA vs. PFA of PVs and LAPW in long-standing persistent AF, and found a similar rate of atrial tachyarrhythmia recurrences (36.7% vs. 40.9%, *p* = 0.680), with a higher rate of periprocedural complications in the hybrid ablation group (12% vs. 0%, *p* = 0.028) [125]. Finally, Bahlke et al. reported a high success rate (freedom from AF/atrial tachycardia in 82%) with catheter ablation of spatiotemporal dispersions (DISPERS) detected by machine learning software in addition to PVI in patients with long-standing persistent AF [126].

## 9. Cardiomyopathies

Patients with coexistent AF and various cardiomyopathies/HF have increased risk of morbidity and mortality [127]. Both the conditions propagate each other, leading to higher symptom burden, recurrent hospitalizations and poor prognosis [128,129,130]. The Catheter Ablation for Atrial Fibrillation in Patients with End-Stage Heart Failure and Eligibility for Heart Transplantation (CASTLE-HTx) trial reported lower risk of composite of all-cause death, left ventricular assist device implantation or urgent heart transplantation with combined catheter ablation and guideline-directed medical therapy, compared to medical therapy alone (8% vs. 30%, *p* < 0.001) [131]. Patients in the CASTLE-HTx trial who had a risk score ≥ 3, derived from predictors of the primary outcome (such as AF burden > 50%, left ventricular EF < 30% and New York Heart Association class ≥ III), had a higher benefit with ablation than those with low risk [132]. Although beneficial, the timing of catheter ablation was debatable in patients with persistent AF and HFrEF, as demonstrated in the study by Zakeri et al., where early routine and delayed selective catheter ablation groups were similar with respect to all-cause death (*p* = 0.731) and combined all-cause death/CV hospitalization (*p* = 0.467) [133]. William et al. reported no additional benefit of LAPW isolation when performed with PVI in patients with HFrEF [134].

Recent meta-analyses have shown the superiority of catheter ablation in both HFrEF and HFpEF [135,136,137,138,139,140]. Bulhões et al. pooled data from eight studies (20,257 patients with AF and HFpEF) showing a significant reduction in risk of death/HF hospitalization (*p* = 0.001), all-cause death (*p* = 0.047), CV death (*p* = 0.014), and HF hospitalization (*p* = 0.011) with catheter ablation as compared to medical therapy [141]. Similarly, in the meta-analysis of six randomized trials (1055 patients with HFrEF) by Pasqualotto et al., catheter ablation was associated with a significant reduction in HF hospitalization, CV mortality, all-cause mortality and AF burden, along with improvement in left ventricular EF and quality of life (*p* < 0.01 for all) [142].

Patients with HCM have abnormal cardiac tissue arrangement and reduced atrial conduction velocity, leading to less effective catheter ablation with increased risk of AF recurrence [143]. In the meta-analysis by Ezzeddine et al., patients with HCM had a higher risk of recurrent atrial arrhythmias after the first AF ablation (RR 1.498, *p* < 0.001), requirement of AADs (RR 2.844, *p* < 0.001), repeat AF ablation (RR 1.544, *p* = 0.02), and recurrent atrial arrhythmias after their last AF ablation (RR 1.607, *p* < 0.001) when compared with those without HCM [144].

## 10. Adverse Events

A recent meta-analysis of 126 studies of AF ablation reported the following to have decreased over time (2000–2018 vs. 2019–2023): serious adverse complications (6.78% vs. 5.16%, *p* < 0.001), procedure-related stroke (0.51% vs. 0.38%, *p* < 0.001), procedure-related vascular complications (2.07% vs. 1.41%, *p* < 0.001), persistent PNI beyond 30 days (0.48% vs. 0.23%, *p* = 0.001), and symptomatic PV stenosis >70% (0.24% vs. 0.07%, *p* < 0.001) [145]. The two time periods had similar rates of procedure-related death (0.05% vs. 0.04%, *p* = 0.792), pericardial effusion requiring intervention (0.82% vs. 0.76%, *p* = 0.606) and esophageal fistula (0.06% vs. 0.06%, *p* = 0.920). Esophageal injury after AF ablation occurred in around 5–40% of patients who underwent RFA, 3–25% with CBA and <10% with high-intensity focused ultrasound [146]. Among the serious complications, esophageal perforation and fistula formation are rare and associated with worse prognosis, with an incidence rate of less than 0.25% with the use of thermal ablation.

Proactive esophageal cooling is one of the approved interventions to reduce the risk of esophageal injury from thermal AF ablation [147]. However, the data from clinical studies has shown varying results. In the meta-analysis of four randomized trials (294 patients) by Hamed et al., esophageal cooling and control groups had no significant differences regarding the incidence of any esophageal injury (15% vs. 19%), mild to moderate esophageal injury (13.6% vs. 12.1%), procedure duration (standardized mean difference [SMD] −0.03), posterior wall RFA time (SMD 0.27), total RFA time (SMD −0.50), and ablation index (SMD 0.16) [148]. Severe esophageal injury was lower in the cooling group (1.5% vs. 9%). The EsophAguS Deviation During RadiofrequencY Ablation of Atrial Fibrillation (EASY AF) trial evaluated a novel device to deviate a segment of the esophagus using vacuum suction and mechanical deflection [149]. The deviation group had a lower rate of ablation injury to the esophageal mucosa (5.7% vs. 35.4%, *p* < 0.0001), severity and number of ablation lesions per patient compared to the control group.

Vascular access-related complications remain the most common adverse events after catheter ablation. Scientific data from randomized and quasi-randomized studies, as well as meta-analyses, consistently demonstrate that ultrasound-guided vascular access reduces complication rates during AF ablation [150]. The 2024 expert consensus document explicitly recommends ultrasound guidance for vascular access in all AF ablations, based on available evidence [29].

Another modality to improve the safety and efficacy of AF ablation is the use of visualizable steerable sheaths which were introduced for real-time visualization inside the 3D-electroanatomical mapping system. In the study by Khalaph et al., patients undergoing ablation using the visualized sheath had a reduction in total procedure duration (108 vs. 112 min, *p* = 0.045), fluoroscopy time (7 vs. 10 min, *p* < 0.001), left atrial dwell time (73 vs. 79 min, *p* = 0.001) and –dose (507 vs. 783 cGy·cm^2^, *p* < 0.001) compared to the matched control group [151].

## 11. Zero-Fluoro AF Ablations

Zero-fluoro AF ablations are part of the routine practice in many centers, particularly in the U.S. [152,153]. Zero-fluoro transseptal puncture is achievable safely, addressing one of the most challenging steps in zero-fluoro PVI procedures [154]. Thus, even without prior relevant experience, fluoroscopy-free catheter ablation can be performed safely [155]. Debreceni et al. conducted a meta-analysis of seven studies (1593 patients) reporting around 95.1% feasibility of the zero-fluoroscopy approach [156]. It led to a significant reduction in procedure time (MD: −9.11 min, *p* < 0.01), fluoroscopy time (MD: −5.21 min, *p* < 0.01), and fluoroscopy dose (MD: −3.96 mGy, *p* < 0.01) when compared with the non-zero fluoroscopy group, without any differences with respect to total ablation time (MD: −104.26 s, *p* = 0.12), acute success rate (RR: 1.01, *p* = 0.72), long-term success rate (RR: 0.96, *p* = 0.56), and complications (RR: 0.94, *p* = 0.89).

## 12. Future Perspectives

The safety and effectiveness of catheter ablation for AF is variable, depending on underlying anatomy, age, gender, presence of comorbidities, use of other medications, physical activity, additional triggers, drivers, and substrates [157,158,159,160,161,162,163,164,165,166,167,168,169,170,171,172,173,174,175,176,177,178]. Same day discharge has become increasingly popular given its low complication rate when compared with overnight stay after AF ablation [179,180]. Upcoming novel techniques for AF ablation include hybrid ablation, EIVOM, stereotactic arrhythmia radioablation (STAR), using cardiac MR to visualize atrial ablation lesions, and transcutaneous vagal nerve stimulation (to prevent recurrence) [181,182,183,184,185,186,187,188,189,190,191].

## 13. Conclusions

Catheter ablation for AF is an ever-growing field, with huge potential for improving patient outcomes in terms of both higher efficacy and fewer adverse events. Future studies, preferably randomized trials, are needed to evaluate the newer advancements in catheter ablation, either alone or combined with other ablative strategies, to reduce patient symptoms and improve quality of life.

## Figures and Tables

**Table 1:** Major ablation methods.

	Radiofrequency Ablation	Cryoballoon Ablation	Pulsed Field Ablation
Energy source	Uses heat energy for controlled focal tissue ablation	Uses cryoenergy to freeze tissue	Uses high-voltage electrical fields for electroporation of the sarcolemmal membrane
Procedure time	Longest	Similar or lesser with Pulsed Field Ablation	
Fluoroscopy dose	Lowest	Similar	
Freedom from atrial tachyarrhythmia recurrence	Usually lower	Similar	
Complications	Low		Higher risk of cardiac tamponade

## Data Availability

The data used in this review paper are available on the online databases.
